# Modulation of Caffeine-Induced Behavioral Alterations by N-Acetylcysteine in a Pharmacological Challenge Model

**DOI:** 10.3390/ph19091419

**Published:** 2026-09-08

**Authors:** Cǎtǎlina Ionescu, Ramona-Alexandra Ciauşu, Mǎlina Visternicu, Viorica Rarinca, Gabriel-Ionut Plavan, Mircea-Nicusor Nicoara, Alin Ciobîcǎ, Andrei Luca, Daniel Timofte, Otilia Novac, Bogdan Novac, Ancuța Andreea Miler, Mihai Hogaş, Simona Hogaş

**Affiliations:** 1 Doctoral School of Biology, Faculty of Biology, “Alexandru Ioan Cuza” University of Iași, 700505 Iasi, Romania; catalinaionescu81@yahoo.com (C.I.); malina.visternicu@yahoo.ro (M.V.); rarinca_viorica@yahoo.com (V.R.); 2 “Ioan Haulica” Institute, Apollonia University, 700511 Iasi, Romania; 3 Doctoral School of Geosciences, Faculty of Geography and Geology, “Alexandru Ioan Cuza” University of Iasi, 700505 Iasi, Romania; ciausuramona05@gmail.com (R.-A.C.); mirmag_02@yahoo.com (M.-N.N.); 4 Department of Biology, Faculty of Biology, Alexandru Ioan Cuza University of Iasi, 700505 Iasi, Romania; gabriel.plavan@uaic.ro; 5 “Olga Necrasov” Center, Department of Biomedical Research, Romanian Academy, 700481 Iasi, Romania; 6 CENEMED Platform for Interdisciplinary Research, University of Medicine and Pharmacy “Grigore T. Popa”, 700115 Iasi, Romania; 7 Faculty of Medicine, “Carol Davila” University of Medicine and Pharmacy, 050474 Bucharest, Romania; zlucaandrei@gmail.com; 8 Grigore T. Popa University of Medicine and Pharmacy Iași, 700115 Iasi, Romania; dantimofte@yahoo.com (D.T.); otilia76@gmail.com (O.N.); mileranca@yahoo.com (A.A.M.); mihaimmh@gmail.com (M.H.); simona.hogas@umfiasi.ro (S.H.)

**Keywords:** caffeine, N-acetylcysteine, zebrafish, anxiety-like behavior, aggression, social preference, oxidative stress

## Abstract

**Background**: Caffeine is a widely consumed psychostimulant that may affect anxiety-like, aggression-like, and social behaviors, potentially in association with oxidative imbalance. **Objectives**: This study evaluated the behavioral and biochemical effects of acute caffeine exposure in adult zebrafish and investigated the potential protective effects of N-acetylcysteine (NAC). **Methods**: Sixty adult wild-type AB zebrafish (*Danio rerio*), approximately 9–10 months old, were assigned to six groups (*n* = 10/group): control, caffeine (25 or 60 mg/L), NAC (1 mg/L), and NAC combined with either caffeine concentration. Behavioral effects were assessed using the Social Preference Test, Mirror-Biting Test, and Novel Tank Test. Superoxide dismutase (SOD) and glutathione peroxidase (GPx) activities were measured as oxidative stress-related markers. **Results**: Caffeine increased locomotor activity and induced anxiety-like and aggression-like behavioral alterations, including increased bottom-dwelling, thigmotaxis, and mirror-directed behavior, while reducing social preference. Caffeine significantly increased SOD activity at 25 mg/L (*p* = 0.010) and 60 mg/L (*p* = 0.008), and GPx activity at 25 mg/L (*p* = 0.008) and 60 mg/L (*p* = 0.006), compared with controls. NAC attenuated caffeine-induced hyperlocomotion and several anxiety-like and aggression-like alterations and modulated antioxidant enzyme responses, but did not fully restore social preference. **Conclusions**: Acute caffeine exposure produces behavioral alterations and changes in antioxidant enzyme activity in adult zebrafish. NAC partially attenuates these effects, particularly hyperlocomotion, anxiety-like behavior, and aggression-like responses, while showing more limited effects on caffeine-induced social alterations.

## 1. Introduction

Caffeine, the primary psychoactive component of coffee, is one of the most widely consumed stimulants of the central nervous system and is well known for its effects on alertness and cognitive performance [1,2,3]. Moderate caffeine intake is generally associated with beneficial neurobehavioral outcomes; its non-selective antagonistic action on adenosine A1 and A2A receptors can enhance dopaminergic activity and neural arousal. In susceptible individuals, these mechanisms may lead to anxiogenic effects, including increased nervousness, heightened physiological arousal, and impaired sleep quality [4,5,6]. At the cellular level, caffeine modulates motor activity, anxiety-related behaviors, glutamatergic signaling, and oxidative processes in the brain through its actions on both neuronal and glial adenosine receptors [7]. Specifically, as a potent neurostimulant chemical, high or chronic doses of caffeine competitively block adenosine receptors, removing the tonic inhibitory control that adenosine normally exerts over central neurotransmission. This blockade triggers a sustained overactivation of excitatory monoaminergic and glutamatergic pathways, leading to an excessive release of glutamate. The resulting overactivation of postsynaptic receptors drives a massive intracellular influx of calcium ions ($Ca^{2+}$), which forces mitochondria into a state of severe bioenergetic stress and abnormally ramps up oxygen consumption [8,9]. This metabolic overexertion overwhelms endogenous antioxidant defenses, leading to a surge in reactive oxygen species (ROS) and reactive nitrogen species (RNS). This cellular oxidative stress damages lipids, proteins, and DNA within key anxiety-regulating neurocircuits, directly translating molecular neurostimulation into behavioral anxiogenesis and physiological reactivity [10,11,12,13].

Beyond its acute stimulatory effects, caffeine is capable of inducing physical dependence, with withdrawal symptoms such as irritability and fatigue [14]. In adolescents, regular caffeine consumption has been associated with increased anxiety, anger, and aggressive behaviors, effects that are likely mediated by sustained physiological arousal and interactions with the brain’s dopaminergic system [15]. These findings suggest that repeated caffeine exposure and withdrawal cycles may exacerbate anxiety-like and aggressive responses, particularly during sensitive developmental periods.

Anxiety disorders are among the most prevalent mental health conditions worldwide and are characterized by excessive and persistent fear or worry that is disproportionate to actual threats and significantly interferes with daily functioning. Despite the availability of pharmacological treatments, current anxiolytic therapies are often limited by adverse side effects and insufficient efficacy in a substantial proportion of patients, underscoring the need for alternative or adjunctive therapeutic strategies [4,16]. Aggression is similarly a multifaceted behavior influenced by individual predispositions and situational factors, such as frustration or provocation. Psychoactive substances, including caffeine, can modulate aggressive responses by increasing the likelihood that aggressive feelings are expressed as overt behavior, with evidence from both animal and human studies indicating that moderate caffeine doses may enhance aggression under provoking or frustrating conditions [17,18,19].

In this context, NAC has gained increasing attention as a potential modulator of anxiety- and aggression-related behaviors. Its antioxidant activity is mainly explained through indirect mechanisms. NAC is deacetylated to cysteine, which serves as a precursor for glutathione (GSH) synthesis, thereby helping to restore intracellular GSH levels, especially under conditions of oxidative depletion. NAC may also promote the formation of hydropersulfides, highly reactive sulfur species with enhanced antioxidant properties [20,21]. NAC is a precursor of glutathione and a potent antioxidant and anti-inflammatory agent that helps maintain cellular redox balance. Through these mechanisms, NAC has demonstrated protective effects against oxidative stress and neuroinflammation, processes closely linked to the development of anxiety-like behaviors, while also supporting neurogenesis and cognitive function [20,22,23]. Furthermore, NAC can partially relieve caffeine-induced neurobehavioral symptoms and oxidative stress by directly counteracting these specific biochemical cascades at multiple levels: first, as a rate-limiting precursor for GSH synthesis, NAC restores intracellular redox homeostasis and directly neutralizes ROS and RNS accumulated during caffeine-driven metabolic hyperactivation [24]. Second, beyond its direct antioxidant properties, NAC normalizes extracellular glutamate levels via the cystine-glutamate antiporter, thereby dampening the excessive glutamatergic hyperactivity, NMDA receptor overactivation, and subsequent excitotoxicity provoked by high caffeine doses [25,26]. Finally, by suppressing pro-inflammatory cytokine expression and mitigating oxidative damage in anxiety-related neurocircuitry (such as areas homologous to the amygdala and hippocampus in vertebrate models like zebrafish), NAC effectively buffers neural circuits against caffeine-driven hyperarousal and behavioral abnormalities [27,28,29,30]. At the organismal level, these neuroprotective and antioxidant actions translate into measurable behavioral recovery. NAC has been shown to reduce the duration of anxiety-like and aggressive behaviors and to enhance social and communicative behaviors in animal models, effects associated with increased glutathione levels, reduced oxidative stress markers such as malondialdehyde, and downregulation of pro-inflammatory cytokines including IL-1 and IL-6 [31].

To counteract oxidative stress, cells rely on a complex antioxidant defense system composed of both enzymatic and non-enzymatic components. Enzymes such as SOD, catalase (CAT), and GPx act sequentially to neutralize ROS, while non-enzymatic antioxidants like reduced GSH, thioredoxin, and melatonin further support redox balance by directly scavenging free radicals [32]. Within this endogenous defense network, reduced GSH plays a critical role as the most abundant intracellular non-enzymatic antioxidant, acting as a direct scavenger of free radicals and serving as an essential cofactor for glutathione peroxidase [33]. Maintaining optimal brain GSH levels is vital for buffering cellular redox homeostasis, protecting lipids, proteins, and DNA from oxidative damage, and preserving normal neurobehavioral functions. Depletion of cerebral GSH significantly compromises cellular resilience, predisposing neural circuits to oxidative stress, neuroinflammation, and excitotoxicity, which directly underlies the manifestation of anxiety-like and stress-related behavioral disturbances [34,35]. Caffeine has been extensively reported to influence extracellular glutamate concentrations and to exert protective effects against oxidative stress in the brain. While both glutamate and ROS are known to contribute to neurobehavioral disturbances, it is still uncertain whether oxidative stress in the brain serves as a biochemical pathway underlying the anxiety-like behaviors associated with caffeine exposure [7].

Zebrafish (*Danio rerio*) are a well-established model organism in behavioral neuroscience due to their genetic tractability, ease of maintenance, and strong translational relevance to human neuropsychiatric conditions [36]. Zebrafish exhibit robust anxiety- and aggression-related behaviors, including freezing, erratic swimming, thigmotaxis, geotaxis, and mirror-biting, which can be quantitatively assessed [37]. Psychoactive compounds such as caffeine can be administered via immersion, enabling both acute and chronic exposure paradigms, while behavioral responses are recorded using video tracking systems to analyze locomotion, freezing, and social or aggressive interactions [38,39,40]. Importantly, behavioral responses to caffeine in zebrafish are modulated by social context, with isolated individuals displaying more pronounced anxiety-like effects, whereas exposure to visual social cues can buffer these responses, emphasizing the role of environmental factors in pharmacological studies [41]. Building on these findings, the zebrafish model can also be effectively used to evaluate the effects of bioactive compounds such as caffeine or antioxidant agents on oxidative stress. Given its well-characterized and reproducible responses to pro-oxidant exposure, zebrafish provide a suitable platform to assess whether caffeine exacerbates or mitigates oxidative imbalance, as well as to test the protective potential of antioxidant treatments. Therefore, zebrafish represent a valuable translational model not only for studying oxidative stress mechanisms but also for screening compounds that may modulate redox homeostasis [42,43].

Based on this evidence, the primary objective of this study was to evaluate the behavioral and biochemical alterations induced by acute pharmacological caffeine exposure in zebrafish, and to investigate whether the administration of N-acetylcysteine can confer neuroprotection by restoring redox balance and mitigating oxidative stress. We hypothesize that pharmacological caffeine exposure will induce anxiety-like and aggressive behaviors in zebrafish, manifested by increased freezing, erratic swimming, and mirror-biting, and that administration of NAC will mitigate these effects through its antioxidant and anti-inflammatory properties, thereby normalizing behavioral responses.

## 2. Results

The Social Preference Test, the Mirror Biting Test and the Novel Tank Test were performed to investigate the potential protective effects of NAC against caffeine-induced alterations, focusing on social interaction, anxiety-related parameters, and aggressive behavior. Furthermore, a biochemical analysis was conducted to evaluate the specific oxidative stress markers (SOD, GPx).

### 2.1. The Social Preference Test

During the social preference test, locomotor parameters were analyzed in the presence of the social stimulus to evaluate behavioral responses under social interaction conditions. Additional results are presented in Supplementary Materials.

**Figure 1:**
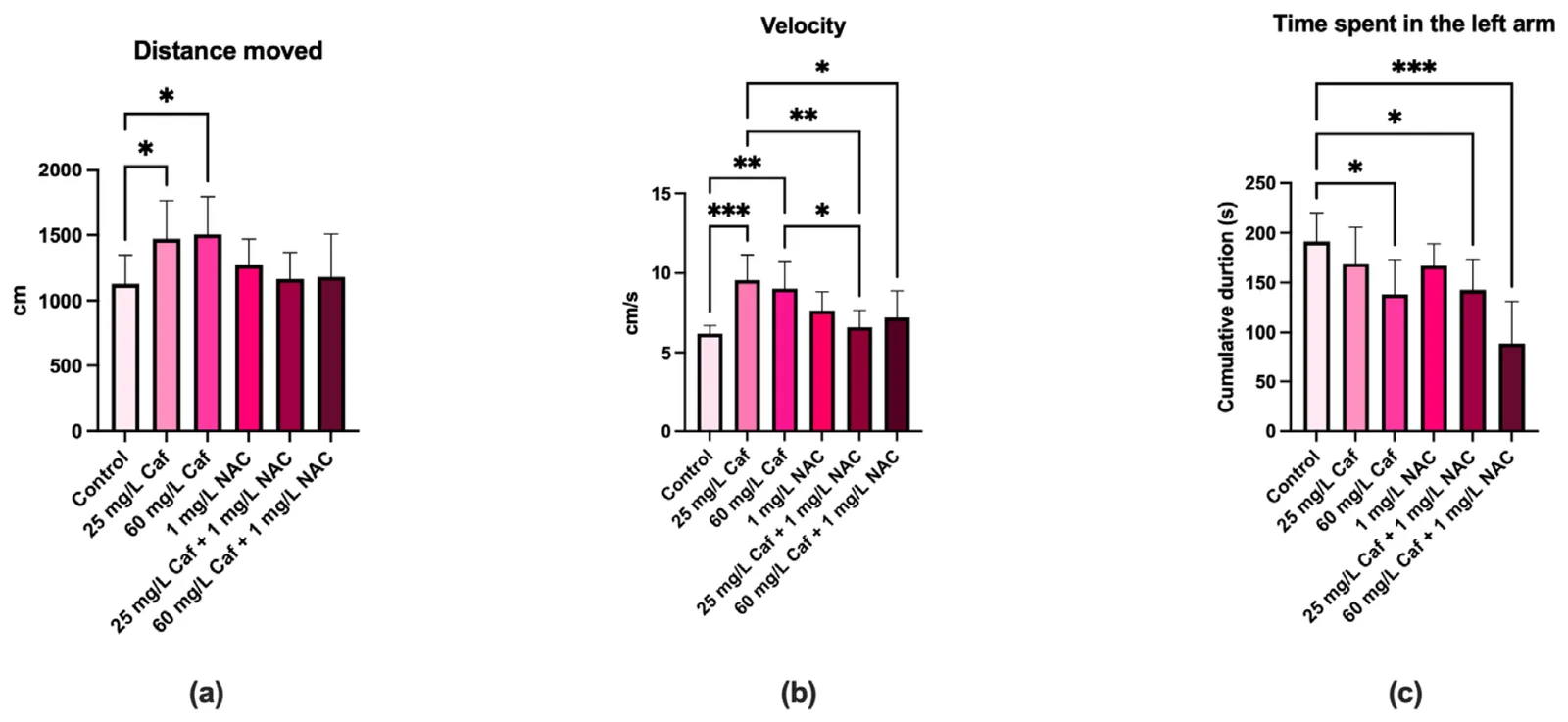
Acute effects of caffeine (25 mg/L and 60 mg/L) alone and in combination with NAC (1 mg/L) on adult zebrafish social interaction. Behavioral parameters measured include: (**a**) total distance moved, (**b**) average velocity, (**c**) time spent in the left arm. Data are presented as mean ± SD (*n* = 10 animals per group, with a 1:1 male-to-female sex ratio) and were analyzed using one-way ANOVA followed by Tukey’s post hoc test. Statistically significant differences are indicated as follows: * *p* < 0.05, ** *p* < 0.01, and *** *p* < 0.001.

The total distance moved was analyzed as a parameter of locomotor activity, in the presence of the social stimulus (Figure 1a). Statistical analysis revealed significant differences between the control group and the 25 mg/L Caf group (*p* = 0.04, ANOVA, Tukey’s post hoc test), as well as between the control group and the 60 mg/L Caf group (*p* = 0.02, ANOVA, Tukey’s post hoc test), indicating that these treatments significantly affected locomotor activity compared to the control group. Although no statistically significant differences were observed for the NAC-treated groups (*p* > 0.05, ANOVA), a decreasing trend in the total distance traveled was noted. A similar reduction in locomotor activity was also observed in the groups receiving NAC combined with 25 mg/L Caf and 60 mg/L Caf; however, these changes did not reach statistical significance.

The average velocity parameter was further analyzed to evaluate locomotor performance (Figure 1b). Significant differences were observed between the control group and 25 mg/L Caf (*p* = 0.001, ANOVA, Tukey’s post hoc test), as well as between the control group and 60 mg/L Caf (*p* = 0.002). In addition, 25 mg/L Caf differed significantly from 25 mg/L Caf + NAC (*p* = 0.001) and from 60 mg/L Caf + NAC (*p* = 0.02). A significant difference was also observed between 60 mg/L Caf and 25 mg/L Caf + NAC (*p* = 0.01). These results indicate that caffeine treatment, alone or in combination with NAC, significantly modulated the locomotor velocity of the zebrafish.

The time spent in the left arm, in the presence of the social stimulus, was analyzed as an indicator of social preference (Figure 1c). Compared to the control group, single caffeine treatments such as 60 mg/L Caf significantly reduced the time spent near the social stimulus (*p* = 0.01, ANOVA, Tukey’s post hoc test). Similarly, the combinations of Caf with NAC (25 mg/L Caf + NAC, *p* = 0.03; 60 mg/L Caf + NAC, *p* < 0.001) also decreased the presence near the social stimulus. These results indicate that both caffeine alone and in combination with NAC attenuate social preference behavior in zebrafish.

**Figure 2:**
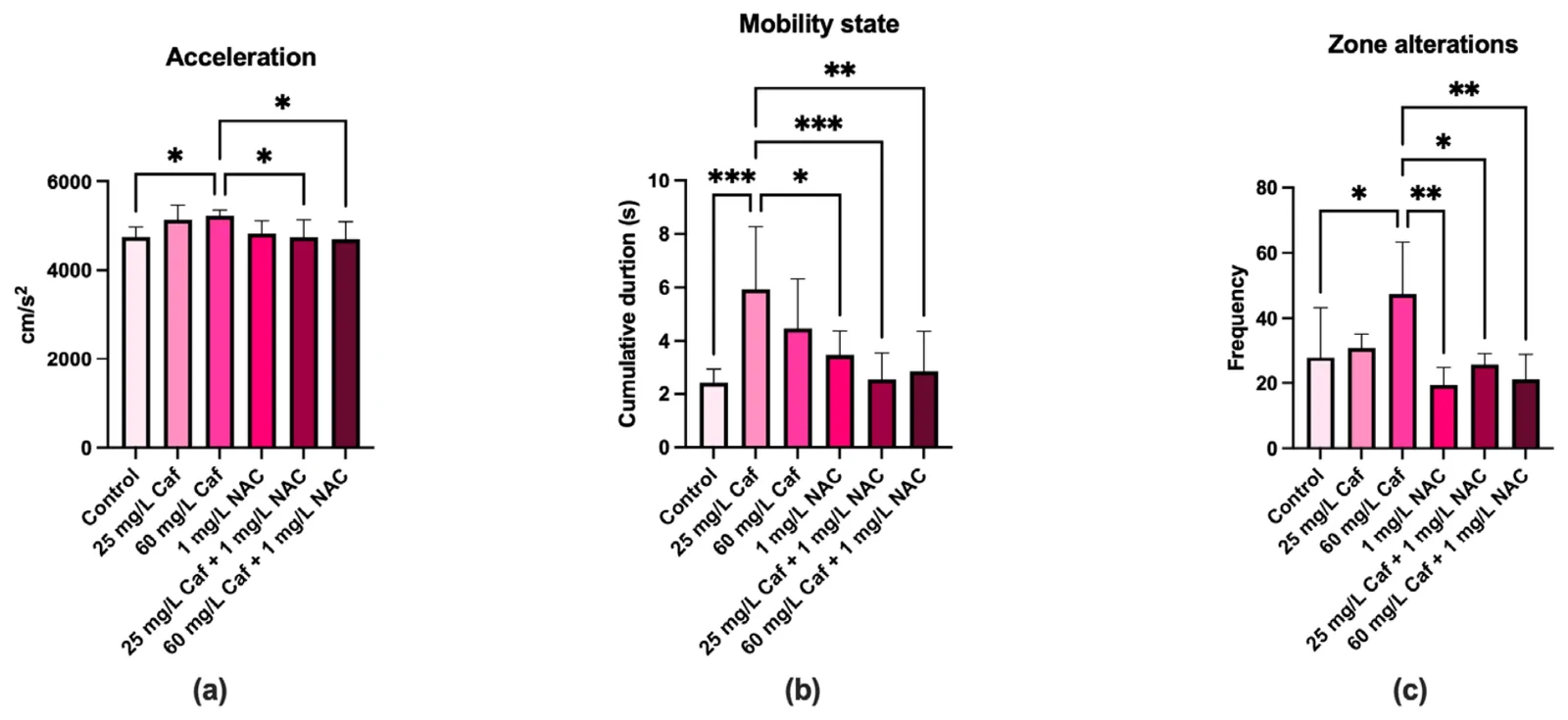
Acute effects of caffeine (25 mg/L and 60 mg/L) alone and in combination with NAC (1 mg/L) on adult zebrafish social interaction. Behavioral parameters measured include: (**a**) acceleration, (**b**) mobility state, and (**c**) zone alterations. Data are presented as mean ± SD (*n* = 10 animals per group, with a 1:1 male-to-female sex ratio) and were analyzed using one-way ANOVA followed by Tukey’s post hoc test. Statistically significant differences are indicated as follows: * *p* < 0.05, ** *p* < 0.01, and *** *p* < 0.001.

Acceleration was also analyzed as a locomotor parameter during the social preference test (Figure 2a). A significant increase in acceleration was observed in 60 mg/L Caf compared to the control group (*p* = 0.04, ANOVA, Tukey’s post hoc test). In contrast, acceleration was significantly decreased in 25 mg/L Caf + NAC compared to 60 mg/L Caf mg/L (*p* = 0.04), as well as in 60 mg/L Caf+ NAC compared to 60 mg/L Caf (*p* = 0.02). These results indicate that NAC, when combined with caffeine, reduces acceleration, counteracting the stimulatory effect of high-dose caffeine.

Mobility state was analyzed as an additional locomotor parameter (Figure 2b). Treatment with 25 mg/L Caf significantly increased mobility compared to the control group (*p* < 0.001, ANOVA, Tukey’s post hoc test). In contrast, mobility was markedly decreased in 25 mg/L Caf + NAC compared to 25 mg/L Caf alone (*p* < 0.001), as well as in NAC alone compared to 25 MG/L CAF (*p* = 0.02), and in 60 mg/L Caf + NAC compared to 25 mg/L Caf (*p* = 0.002). These results indicate that NAC, either alone or in combination with caffeine, reduces mobility, counteracting the stimulatory effects of 25 mg/L Caf.

Zone alterations, the last parameter analyzed during the social preference test, were significantly affected by caffeine and NAC treatments (Figure 2c). Treatment with 60 mg/L Caf significantly increased zone alterations compared to the control group (*p* = 0.04, ANOVA, Tukey’s post hoc test). In contrast, NAC alone significantly decreased zone alterations compared to 60 mg/L Caf (*p* = 0.005). Similarly, 25 mg/L Caf + NAC (*p* = 0.05) and 60 mg/L Caf + NAC (*p* = 0.010) also reduced zone alterations compared to 60 mg/L Caf. These findings indicate that NAC, either alone or in combination with caffeine, attenuates the stimulatory effect of high-dose caffeine on exploratory behavior.

### 2.2. The Mirror-Biting Test

To assess aggressive behavior, locomotor and biting responses were measured during the mirror biting test in the presence of a mirror stimulus, allowing evaluation of behavioral reactions under aggression-like conditions. Additional results are presented in Supplementary Materials.

**Figure 3:**
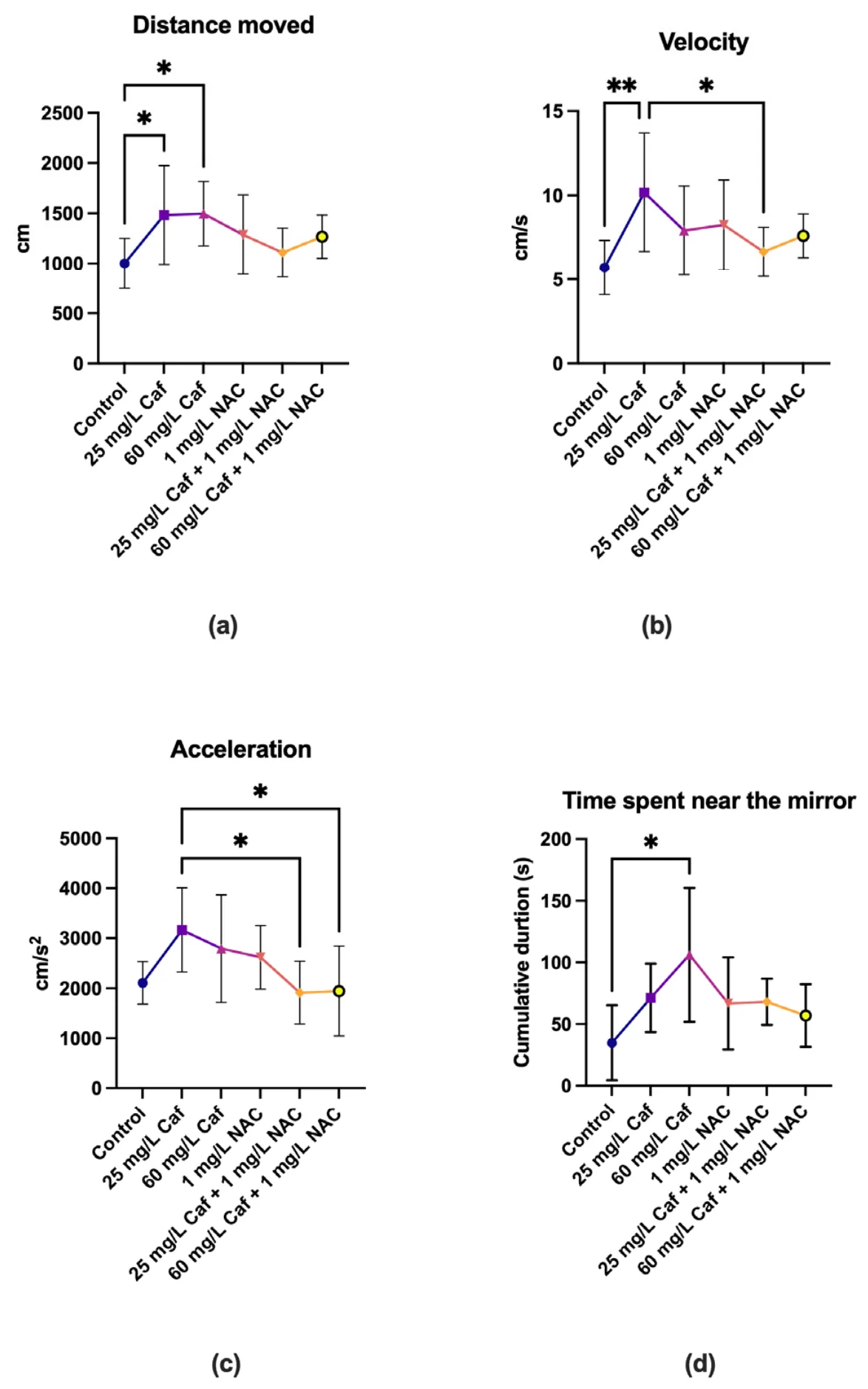
Acute effects of caffeine (25 mg/L and 60 mg/L) alone and in combination with NAC (1 mg/L) on adult zebrafish aggressive-like behavior in the mirror biting test. Behavioral parameters measured include: (**a**) total distance moved, (**b**) average velocity, (**c**) acceleration, (**d**) time spent near the mirror. Single caffeine treatments enhanced locomotor and aggressive behaviors, whereas NAC, either alone or in combination with caffeine, attenuated these effects. Data are presented as mean ± SD (*n* = 10 animals per group, with a 1:1 male-to-female sex ratio) and were analyzed using one-way ANOVA followed by Tukey’s post hoc test. Statistically significant differences are indicated as follows: * *p* < 0.05, and ** *p* < 0.01.

The total distance moved was analyzed as a locomotor parameter during the mirror biting test (Figure 3a). Treatment with 25 mg/L Caf and 60 mg/L Caf significantly increased the total distance moved compared to the control group (25 mg/L Caf, *p* = 0.04; 60 mg/L Caf, *p* = 0.03, ANOVA, Tukey’s post hoc test), indicating a stimulatory effect of caffeine on locomotor activity in the presence of the mirror stimulus.

Average velocity was analyzed as an additional locomotor parameter during the mirror biting test (Figure 3b). Treatment with 25 mg/L Caf significantly increased average velocity compared to the control group (*p* = 0.005, ANOVA, Tukey’s post hoc test), indicating heightened locomotor and aggressive activity. In contrast, pre-treatment with NAC in the 25 mg/L Caf + NAC group attenuated this increase in velocity, suggesting a modulatory effect on caffeine-induced aggressive behavior.

Acceleration was also analyzed during the mirror biting test to further characterize locomotor activity (Figure 3c). Treatment with 25 mg/L Caf significantly increased acceleration compared to the control group, indicating heightened locomotor and aggressive activity. In contrast, pre-treatment with NAC in the 25 mg/L Caf + NAC group significantly decreased acceleration compared to 25 mg/L Caf alone (*p* = 0.03, ANOVA, Tukey’s post hoc test). Similarly, 60 mg/L Caf + NAC also showed a significant decrease in acceleration compared to 25 mg/L Caf (*p* = 0.03), demonstrating that NAC attenuates caffeine-induced increases in locomotor and aggressive-like responses.

Time spent near the mirror was measured to assess aggressive behavior during the mirror biting test (Figure 3d). Treatment with 60 mg/L Caf significantly increased the time spent near the mirror compared to the control group (*p* = 0.01, ANOVA, Tukey’s post hoc test), indicating heightened aggressive-like behavior in zebrafish. Although no other statistically significant differences were observed, groups pre-treated with NAC tended to spend less time near the mirror, suggesting an attenuation of caffeine-induced aggressive behavior.

**Figure 4:**
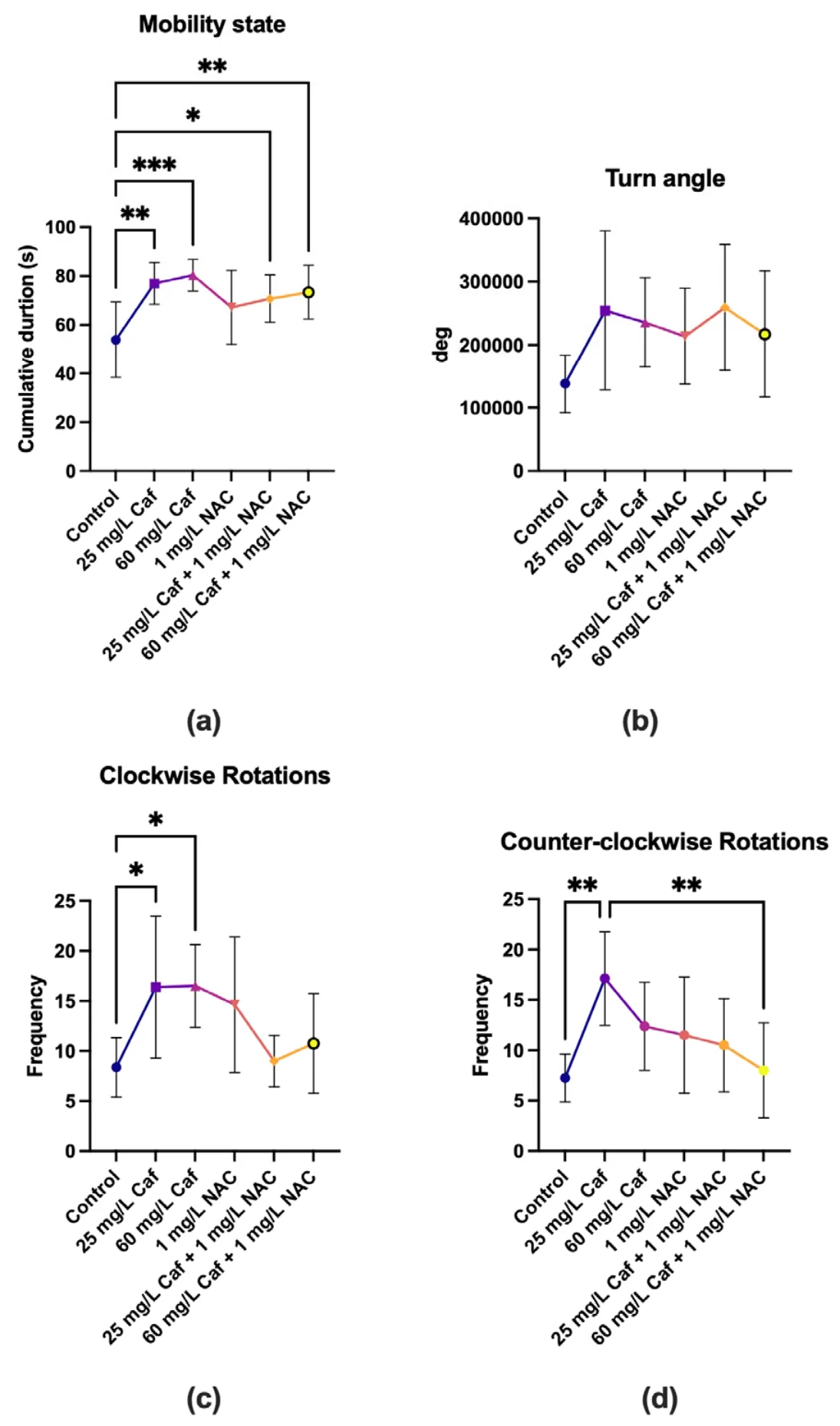
Acute effects of caffeine (25 mg/L and 60 mg/L) alone and in combination with NAC (1 mg/L) on adult zebrafish aggressive-like behavior in the mirror biting test. Behavioral parameters measured include: (**a**) mobility state, (**b**) turn angle, (**c**) clockwise rotations, and (**d**) counterclockwise rotations. Single caffeine treatments enhanced locomotor and aggressive behaviors, whereas NAC, either alone or in combination with caffeine, attenuated these effects. Data are presented as mean ± SD (*n* = 10 animals per group, with a 1:1 male-to-female sex ratio) and were analyzed using one-way ANOVA followed by Tukey’s post hoc test. Statistically significant differences are indicated as follows: * *p* < 0.05, ** *p* < 0.01, and *** *p* < 0.001.

Mobility state was analyzed to evaluate overall locomotor and aggressive behavior during the mirror biting test (Figure 4a). Treatment with 25 mg/L Caf and 60 mg/L Caf significantly increased mobility compared to the control group (25 mg/L Caf, *p* = 0.001; 60 mg/L Caf, *p* < 0.001, ANOVA, Tukey’s post hoc test), reflecting enhanced locomotor and aggressive activity. Treatment with 25 mg/L Caf + NAC and 60 mg/L Caf + NAC also showed a significant increase in mobility compared to control (25 mg/L Caf + NAC, *p* = 0.03; 60 mg/L Caf + NAC, *p* = 0.008), but the effect was smaller than that observed in the caffeine-only groups. These observations suggest that NAC partially attenuates the stimulatory effects of caffeine on locomotor and aggressive behavior, while some enhancement in mobility persists.

Turn angle was analyzed to further assess locomotor patterns during the mirror biting test (Figure 4b). Although no statistically significant differences were observed, several trends were noted: 25 mg/L Caf showed an increase in turn angle compared to control, while 60 mg/L Caf exhibited a slight decrease but remained higher than the control. NAC alone also showed a small increase in turn angle, and 25 mg/L Caf + NAC displayed an increase, whereas 60 mg/L Caf + NAC demonstrated a tendency toward decreased turn angle. These observations suggest subtle modulations in locomotor patterns depending on caffeine and NAC treatments, even in the absence of statistical significance.

Clockwise rotations were analyzed to evaluate directional locomotor patterns during the mirror biting test (Figure 4c). The 25 mg/L Caf and 60 mg/L Caf treatments significantly increased clockwise rotations compared to the control group (25 mg/L Caf, *p* = 0.01; 60 mg/L Caf, *p* = 0.01, ANOVA, Tukey’s post hoc test), indicating enhanced locomotor and aggressive activity. In the remaining groups, a trend toward decreased clockwise rotations was observed, suggesting subtle modulatory effects of NAC and caffeine combinations on directional movement.

To further examine directional locomotor activity during the mirror biting test, counterclockwise rotations were measured (Figure 4d). 25 mg/L Caf significantly increased counterclockwise rotations compared to the control group (*p* = 0.001, ANOVA, Tukey’s post hoc test), reflecting heightened locomotor and aggressive responses. In contrast, 60 mg/L Caf + NAC showed a significant decrease in counterclockwise rotations compared to 25 mg/L Caf (*p* = 0.003), indicating that NAC attenuates caffeine-induced enhancement of directional locomotor activity.

### 2.3. The Novel Tank Test

To assess anxiety-like behavior, locomotor activity, vertical exploration, and thigmotaxis were measured during the Novel Tank Test, allowing evaluation of behavioral responses under anxiogenic conditions. Vertical exploration (time spent in top versus bottom zones) and preference for the tank periphery provide complementary measures of anxiety-like behavior in zebrafish. Additional results are presented in Supplementary Materials.

**Figure 5:**
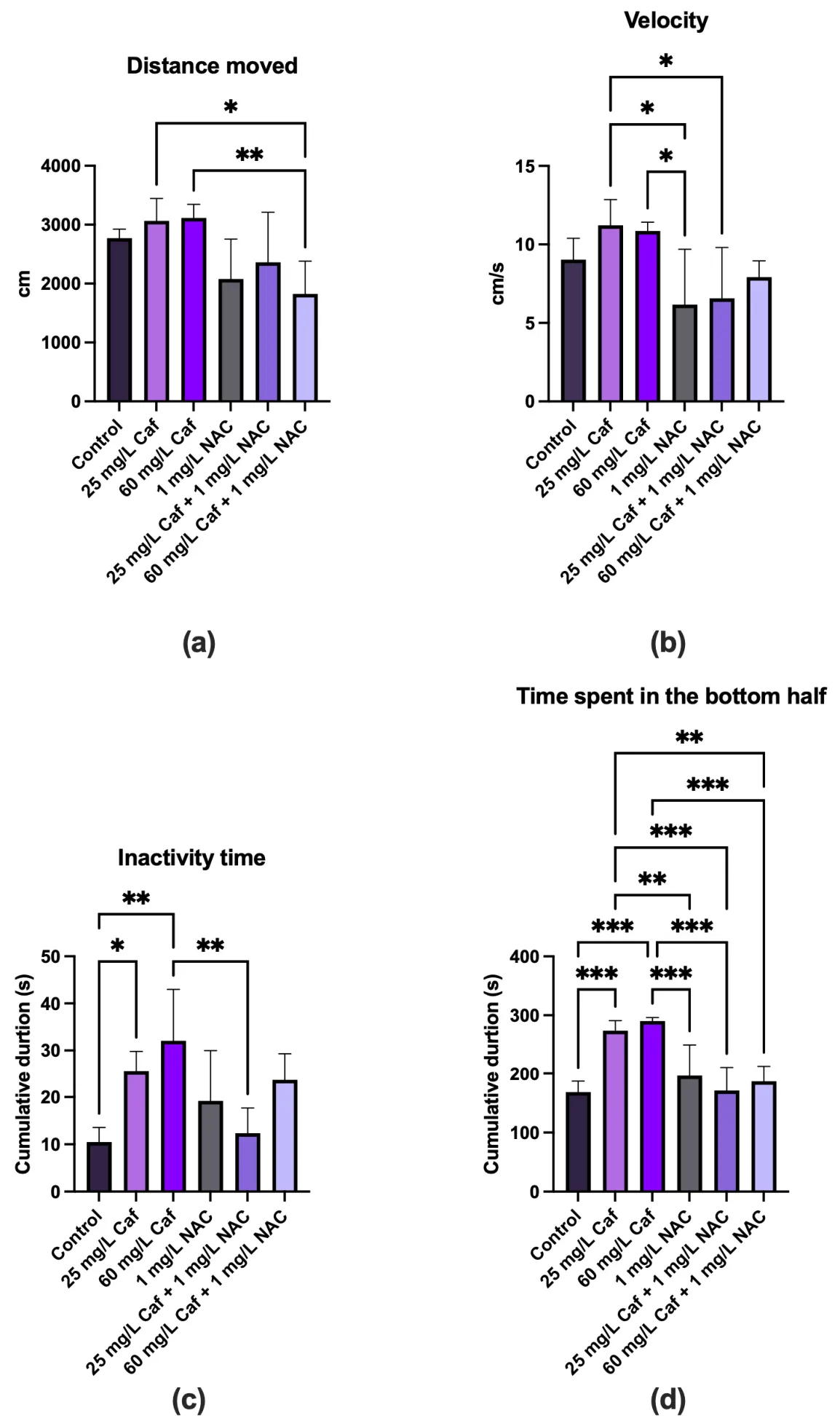
Acute effects of caffeine (25 mg/L and 60 mg/L) alone and in combination with NAC (1 mg/L) on adult zebrafish anxiety-like behavior in the Novel Tank Test. Behavioral parameters measured include: (**a**) total distance moved, (**b**) average velocity, (**c**) inactivity time, (**d**) time spent in the bottom half. Single caffeine treatments increased locomotor activity and anxiety-like behavior, whereas NAC, either alone or in combination with caffeine, attenuated these effects. Data are presented as mean ± SD (*n* = 10 animals per group, with a 1:1 male-to-female sex ratio) and were analyzed using one-way ANOVA followed by Tukey’s post hoc test. Statistically significant differences are indicated as follows: * *p* < 0.05, ** *p* < 0.01, and *** *p* < 0.001.

Total distance moved was analyzed to assess locomotor activity during the Novel Tank Test (Figure 5a). A significant decrease in total distance moved was observed in the 60 mg/L Caf + NAC group compared to 25 mg/L Caf (*p* = 0.01, ANOVA, Tukey’s post hoc test), and similarly, 60 mg/L Caf + NAC showed a significant decrease compared to 60 mg/L Caf alone (*p* = 0.01). These results suggest that NAC attenuates caffeine-induced hyperlocomotion in zebrafish under anxiogenic conditions.

To further evaluate locomotor activity, average velocity was assessed (Figure 5b). Pre-treatment with NAC significantly decreased velocity compared to 25 mg/L Caf (*p* = 0.01) and 60 mg/L Caf (*p* = 0.03, ANOVA, Tukey’s post hoc test), indicating that NAC attenuates caffeine-induced hyperactivity. Additionally, 25 mg/L Caf + NAC showed a significant decrease in velocity compared to 25 mg/L Caf alone (*p* = 0.03), further supporting the modulatory effect of NAC on caffeine-stimulated locomotor activity under anxiogenic conditions.

To further characterize anxiety-like behavior in the Novel Tank Test, inactivity time was measured (Figure 5c). Treatment with 25 mg/L Caf significantly increased inactivity time compared to the control (*p* = 0.03), while 60 mg/L Caf caused an even greater increase (*p* = 0.002), indicating heightened anxiety-like behavior. In contrast, 60 mg/L Caf vs. 25 mg/L Caf + NAC showed a decrease in inactivity time, reflecting increased locomotor activity and suggesting that NAC attenuates caffeine-induced anxiogenic effects.

Time spent in the bottom half of the tank, a key indicator of anxiety-like behavior in zebrafish, was evaluated during the Novel Tank Test (Figure 5d). The 25 mg/L Caf and 60 mg/L Caf treatments significantly increased time spent in the bottom half compared to control (25 mg/L Caf, *p* < 0.001; 60 mg/L Caf, *p* < 0.001, ANOVA, Tukey’s post hoc test), indicating heightened anxiety-like behavior. Pre-treatment with NAC, either alone or in combination with caffeine, significantly decreased time spent in the bottom half compared to both 25 mg/L Caf and 60 mg/L Caf groups (25 mg/L Caf vs. NAC, *p* = 0.007; 25 mg/L Caf vs. 25 mg/L Caf + NAC, *p* < 0.001; 25 mg/L Caf vs. 60 mg/L Caf + NAC, *p* = 0.002; 60 mg/L Caf vs. NAC, *p* < 0.001; 60 mg/L Caf vs. 25 mg/L Caf + NAC, *p* < 0.001; 60 mg/L Caf vs. 60 mg/L Caf + NAC, *p* < 0.001), demonstrating that NAC effectively attenuates caffeine-induced anxiety-like behavior.

**Figure 6:**
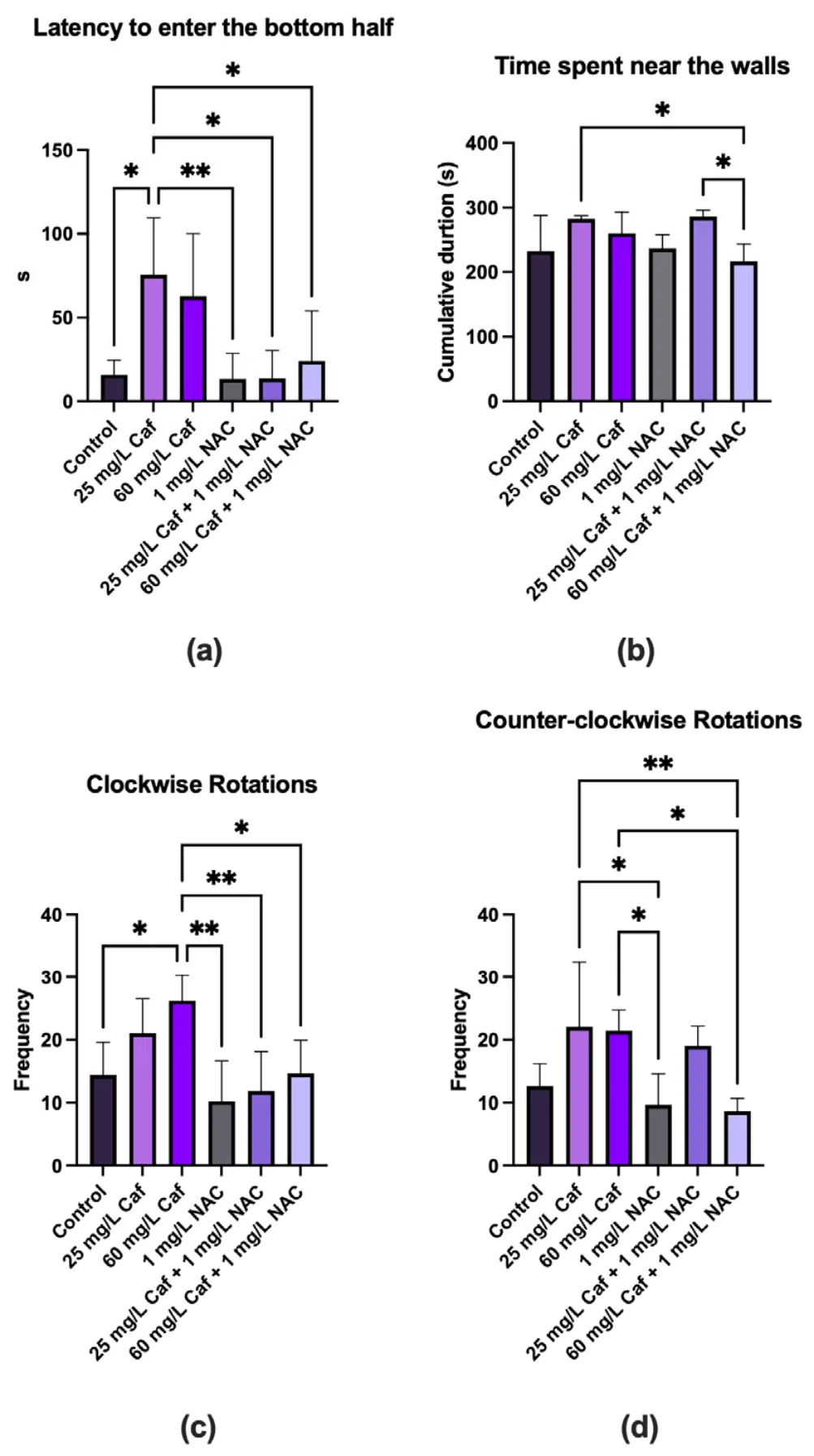
Acute effects of caffeine (25 mg/L and 60 mg/L) alone and in combination with NAC (1 mg/L) on adult zebrafish anxiety-like behavior in the Novel Tank Test. Behavioral parameters measured include: (**a**) latency to enter the bottom half, (**b**) thigmotaxis (time spent near the walls), (**c**) clockwise rotations, and (**d**) counterclockwise rotations. Single caffeine treatments increased locomotor activity and anxiety-like behavior, whereas NAC, either alone or in combination with caffeine, attenuated these effects. Data are presented as mean ± SD (*n* = 10 animals per group, with a 1:1 male-to-female sex ratio) and were analyzed using one-way ANOVA followed by Tukey’s post hoc test. Statistically significant differences are indicated as follows: * *p* < 0.05, and ** *p* < 0.01.

Latency to enter the bottom half was measured as an indicator of anxiety-like exploratory behavior as well (Figure 6a). Treatment with 25 mg/L Caf significantly increased latency to enter to the bottom half compared to control (*p* = 0.01, ANOVA, Tukey’s post hoc test), reflecting heightened anxiety and defensive exploration. Pre-treatment with NAC, either alone or combined with caffeine, significantly decreased this parameter compared to 25 mg/L Caf (25 mg/L Caf vs. 25 mg/L Caf + NAC, *p* = 0.01; 25 mg/L Caf vs. NAC, *p* = 0.010; 25 mg/L Caf vs. 60 mg/L Caf + NAC, *p* = 0.05), indicating that NAC attenuates caffeine-induced anxiety-like behavior by reducing defensive bottom-zone exploration.

Thigmotaxis, measured as the time spent near the tank walls, was analyzed as an additional indicator of anxiety-like behavior (Figure 6b). Treatment with 25 mg/L Caf + NAC and 25 mg/L Caf alone both showed a decrease in time spent near the walls when compared to 60 mg/L Caf + NAC (25 mg/L Caf vs. 60 MG/L CAF + NAC, *p* = 0.02; 25 mg/L Caf + NAC vs. 60 mg/L Caf + NAC, *p* = 0.01, ANOVA, Tukey’s post hoc test), suggesting that NAC reduces anxiety-like behavior by promoting exploration of the central areas of the tank.

Clockwise rotations were analyzed to assess directional locomotor activity (Figure 6c). Treatment with 60 mg/L Caf significantly increased clockwise rotations compared to the control group (*p* = 0.03, ANOVA, Tukey’s post hoc test), indicating heightened locomotor activity under anxiogenic conditions. Pre-treatment with NAC, either alone or in combination with caffeine, significantly decreased clockwise rotations compared to 60 mg/L Caf (60 mg/L Caf vs. NAC, *p* = 0.002; 60 mg/L Caf vs. 25 mg/L Caf + NAC, *p* = 0.005; 60 mg/L Caf vs. 60 mg/L Caf + NAC, *p* = 0.03), demonstrating that NAC attenuates caffeine-induced increases in directional locomotor activity associated with anxiety-like behavior.

Counterclockwise rotations were measured to further evaluate directional locomotor activity under anxiogenic conditions (Figure 6d). Significant decreases were observed in groups pre-treated with NAC, either alone or in combination with caffeine, compared to caffeine-only groups: 25 mg/L Caf vs. NAC, *p* = 0.01; 25 mg/L Caf vs. 60 mg/L Caf + NAC, *p* = 0.007; 60 mg/L Caf vs. NAC, *p* = 0.02; and 60 mg/L Caf vs. 60 mg/L Caf + NAC, *p* = 0.01 (ANOVA, Tukey’s post hoc test). These findings indicate that NAC effectively attenuates caffeine-induced enhancements in directional locomotor activity associated with anxiety-like behavior.

### 2.4. Oxidative Stress Levels

**Figure 7:**
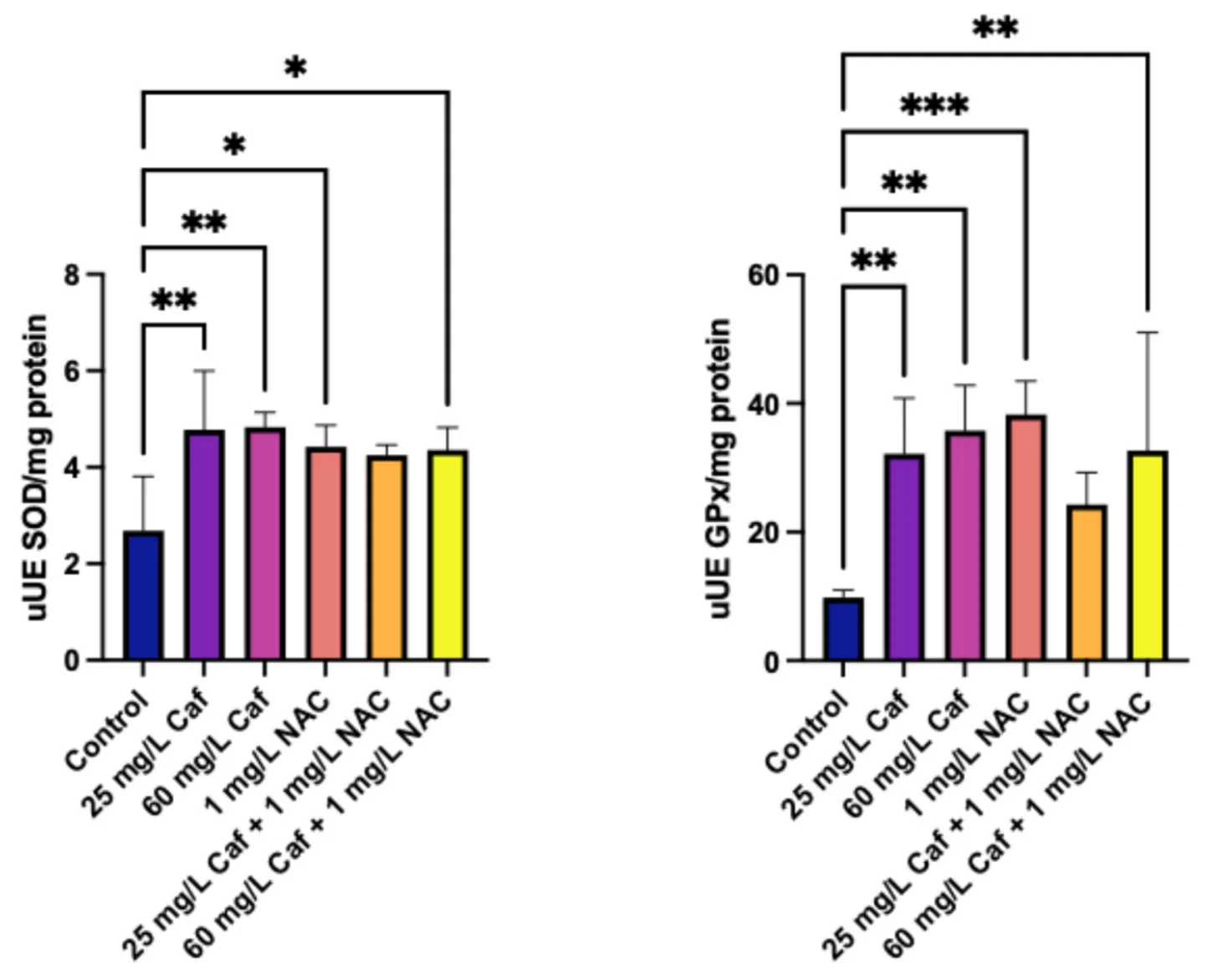
The graphic representation of the oxidative stress marker SOD (uUE SOD/mg protein) and GPx (uUE GPx/mg protein). Data are presented as mean ± SD (*n* = 10 animals per group, with a 1:1 male-to-female sex ratio) and were analyzed using one-way ANOVA followed by Tukey’s post hoc test. Statistically significant differences are indicated as follows: * *p* < 0.05, ** *p* < 0.01, *** *p* < 0.001.

The biochemical analysis of SOD activity is shown in Figure 7. Significant differences were observed among the experimental groups. Compared to the control, SOD activity was significantly increased in the 25 mg/L Caf group (*p* = 0.010) and the 60 mg/L Caf group (*p* = 0.008). Treatment with NAC alone also resulted in a significant change compared to control (*p* = 0.04), while co-treatment with NAC and 60 mg/L Caf showed a borderline significant difference versus control (*p* = 0.05, Tukey, ANOVA). These findings indicate that caffeine modulates SOD activity in a dose-dependent manner, and NAC may influence enzymatic antioxidant responses when administered alone or in combination with caffeine.

The biochemical analysis of GPx activity is presented also in Figure 7. Statistically significant differences were observed among the experimental groups when compared to the control. GPx activity was significantly increased in the 25 mg/L Caf group (*p* = 0.008) and in the 60 mg/L Caf group (*p* = 0.006), indicating a dose-dependent enhancement of antioxidant enzymatic activity following caffeine exposure. The NAC-treated group exhibited a highly significant increase in GPx activity compared to control (*p* = 0.005), suggesting a strong stimulatory effect of NAC on the glutathione-dependent antioxidant system. Similarly, the group co-treated with 60 mg/L Caf and NAC showed a significant elevation in GPx levels (*p* = 0.007) relative to control.

These results suggest that both caffeine and NAC enhance GPx activity, with NAC exerting a more pronounced effect. The observed increase in GPx activity may reflect an adaptive cellular response to oxidative stress, characterized by upregulation of enzymatic antioxidant defenses. Furthermore, the combined treatment appears to maintain elevated GPx activity, indicating that NAC may support or potentiate antioxidant responses in the presence of caffeine-induced oxidative challenges.

## 3. Discussion

Previous research has demonstrated that caffeine exposure can induce neurobehavioral alterations, including hyperlocomotion, increased anxiety-like behavior, and reduced social affiliation, which have been proposed in the literature to involve adenosinergic and dopaminergic pathway modulation as well as oxidative stress-related processes, although these mechanisms were not directly assessed in the present study [7,41,44,45].

The present study demonstrates that acute caffeine exposure induces significant alterations across multiple behavioral domains in adult zebrafish, including locomotor activity, social preference, aggression-like responses, and anxiety-like behavior, alongside changes in antioxidant enzyme activity. Pre-treatment with NAC attenuated several of these caffeine-induced effects, particularly hyperlocomotion, anxiety-like, and aggression-like responses. However, NAC did not uniformly reverse all behavioral alterations, as the impairment in social preference persisted. This differential response suggests that caffeine-induced behavioral alterations may involve partially distinct neurobiological processes, with some being more responsive to antioxidant modulation than others.

In the Social Preference Test, caffeine exposure at 25 mg/L and 60 mg/L significantly impaired social interaction. Zebrafish spent less time in the left arm near the social stimulus, indicating reduced social affiliation. Interestingly, these same fish exhibited increased total distance moved and average velocity, suggesting heightened locomotor activity alongside diminished social engagement. This pattern reflects a state of restless exploration or hyperarousal, in which fish are more active but engage less with conspecifics. In other words, although zebrafish moved more, they spent less time near the social stimulus. Notably, both our study and Wilson et al. 2024 [41] observed common effects of caffeine, including increased locomotor activity and reduced social interaction, highlighting a consistent pattern of hyperactive yet socially withdrawn behavior, although the buffering influence of social cues reported in their study contrasts with the limited restoration of social preference seen with NAC treatment in ours. Similarly, the study by Nair et al. 2025 [46] reported that acute caffeine exposure can both enhance locomotion and reduce social preference, further supporting the idea that caffeine simultaneously increases excitability while impairing social affiliation. This pattern reflects a state of restless exploration or heightened activation, in which fish are more active but engage less with conspecifics. Interestingly, these behavioral changes align with findings from ref. [47], which demonstrated that acute caffeine exposure increases locomotor activity while reducing time spent near social stimuli, highlighting a consistent dose-dependent disruption of social preference. Together, our results and those from the modeling study support the notion that caffeine induces hyperactive yet socially withdrawn behavior in zebrafish, although the underlying neurochemical mechanisms remain speculative in the absence of direct measurements.

Although NAC pre-treatment effectively attenuated caffeine-induced increases in distance moved, velocity, acceleration, and mobility, it did not significantly restore the time spent near the social stimulus in the Social Preference Test. This suggests that while NAC can reduce hyperlocomotion and erratic exploratory behavior, its antioxidant and anti-inflammatory effects may be insufficient to fully prevent caffeine-induced social avoidance. In other words, NAC can normalize general locomotor hyperactivity, but the neural circuits specifically mediating social affiliation cannot be inferred from the present data and were not directly investigated. This dissociation highlights that locomotor stabilization and social behavior recovery are not always coupled, indicating that caffeine-induced reductions in social presence may involve additional mechanisms, such as modulation of social reward pathways, that NAC cannot fully compensate for. In our study, although NAC effectively attenuated hyperactivity and erratic exploratory behavior in zebrafish, it did not restore the time spent near the social stimulus, suggesting that caffeine’s impact on social affiliation may involve neural circuits not fully responsive to NAC. In contrast, in a mouse model of social isolation stress, NAC treatment successfully improved social interaction, increasing the duration of communication behaviors that had been suppressed by isolation [31]. This comparison suggests that the efficacy of NAC in restoring social behavior may depend on the type of social deficit and the underlying neurobiological mechanisms, with caffeine-induced social avoidance in zebrafish potentially involving pathways less sensitive to antioxidant modulation. Moreover, a study by Wang et al. 2024 [48] reported that NAC improved social deficits and oxidative stress-related alterations in a zebrafish model of arsenic exposure, suggesting that NAC may exert beneficial effects on social behavior under certain neurotoxic conditions.

Human studies support a link between caffeine and aggression. In a large adult sample including both healthy and aggressive individuals, higher caffeine intake was associated with increased aggression, anger, and impulsivity, even when controlling for psychiatric comorbidities. This aligns with our zebrafish findings, where acute caffeine exposure enhanced locomotor and aggression-like behaviors, suggesting that caffeine can promote hyperactive and aggressive responses across species [49,50]. In our study, caffeine exposure markedly increased aggression-related parameters in the Mirror-biting Test. Both 25 mg/L and 60 mg/L caffeine significantly elevated total distance moved, velocity, acceleration, and mobility, while also increasing time spent near the mirror and clockwise/counter-clockwise rotations. These findings indicate heightened locomotor and aggressive-like responses in the presence of a provocative social stimulus. Pre-treatment with NAC attenuated these stimulatory and aggressive effects, with reductions in acceleration, mobility, and directional rotations compared to caffeine-alone groups. Notably, time spent near the mirror decreased in NAC + caffeine groups, suggesting partial normalization of aggressive tendencies. These results support the notion that caffeine-induced aggression may be mediated, at least in part, by oxidative stress and hyperactivation of neural circuits regulating arousal and motivation, which NAC can mitigate through its antioxidant activity [17,18]. These results support the notion that caffeine-induced aggression-like responses may be associated with changes in arousal-related processes and antioxidant balance, although a causal role of oxidative stress cannot be established based on the present data.

Previous studies using zebrafish have demonstrated that acute exposure to anxiogenic stimuli can significantly alter vertical exploration, thigmotaxis, and locomotor activity, highlighting the sensitivity of zebrafish to environmental and pharmacological stressors [51,52,53]. In the Novel Tank Test, caffeine showed clear anxiogenic effects. Zebrafish exposed to caffeine spent more time in the bottom half of the tank, displayed increased transitions to the bottom, and showed elevated thigmotaxis, consistent with heightened anxiety-like behavior. Additionally, locomotor parameters such as total distance moved, velocity, and clockwise and counter-clockwise rotations were increased, reflecting hyperactivity under anxiogenic conditions. NAC pre-treatment attenuated many of these effects, significantly decreasing bottom-dwelling, transitions, thigmotaxis, and directional rotations. Locomotor hyperactivity was also attenuated, with reductions in distance moved, velocity, and acceleration compared to caffeine-only groups. Collectively, these findings indicate that NAC can buffer caffeine-induced anxiety-like behaviors by restoring exploratory patterns, reducing hyperlocomotion, and modulating spatial preferences. The convergence of several behavioral endpoints strengthens the interpretation of an anxiety-like response rather than an isolated alteration in swimming activity. In particular, changes in bottom-related exploration and thigmotaxis occurred alongside alterations in locomotor and directional parameters. The partial normalization of several of these endpoints following NAC pre-treatment further supports a modulatory effect of NAC on the behavioral response to caffeine. Nevertheless, because locomotor activity can influence the expression of anxiety-related endpoints in zebrafish, these findings should be interpreted as evidence of anxiety-like behavioral modulation rather than as a direct measure of an underlying emotional state.

Biochemical analyses of SOD activity confirmed that caffeine exposure increased oxidative stress in a dose-dependent manner. Although NAC is a glutathione precursor and a potent antioxidant, SOD activity remained elevated in NAC-treated groups, either alone or in combination with caffeine, albeit at lower levels than in the caffeine-only groups. This partial elevation likely reflects a compensatory activation of the endogenous antioxidant defense system rather than persistent oxidative damage. NAC does not completely suppress ROS production but instead enhances redox buffering capacity by supporting glutathione synthesis and restoring cellular homeostasis. Therefore, the moderate increase in SOD activity observed in NAC-treated groups may indicate an adaptive antioxidant response aimed at maintaining redox balance under pharmacological challenge [7,32].

A similar pattern was observed in the case of GPx activity. Caffeine exposure led to a significant increase in GPx levels at both tested concentrations, further supporting the presence of oxidative stress and the activation of enzymatic antioxidant defenses. NAC administration resulted in a pronounced elevation of GPx activity, consistent with its role as a precursor of reduced glutathione, which is essential for GPx function. In the combined treatment group, GPx activity remained significantly elevated, suggesting that NAC enhances the glutathione-dependent detoxification pathway even in the presence of caffeine-induced oxidative stimuli. Unlike SOD, which primarily catalyzes the dismutation of superoxide radicals, GPx directly reduces hydrogen peroxide and lipid peroxides, indicating that the antioxidant response involves multiple complementary pathways. The sustained increase in GPx activity across treated groups likely reflects an adaptive mechanism aimed at limiting oxidative damage and maintaining cellular redox equilibrium rather than indicating excessive oxidative injury.

Regarding the observation that SOD and GPx activities did not fully return to baseline levels following NAC treatment, this may be partly explained by the pharmacological action of NAC itself. As a precursor that supports de novo glutathione synthesis, exogenous NAC may sustain glutathione-dependent antioxidant defenses. Rather than necessarily returning antioxidant enzyme activities to basal levels, NAC may enhance the tissue’s overall redox-buffering capacity, resulting in a sustained adaptive antioxidant response that contributes to maintaining cellular redox homeostasis under pharmacological challenge.

When the biochemical and behavioral findings are considered together, a parallel pattern emerges in which caffeine-induced behavioral alterations were accompanied by changes in antioxidant enzyme activity, while NAC treatment modulated both behavioral responses and antioxidant defenses. This correspondence is consistent with the potential involvement of redox-sensitive processes in the response to acute caffeine exposure. However, the present data do not establish a direct causal relationship between oxidative changes and individual behavioral outcomes, because SOD and GPx activities were measured as endpoint biochemical markers and were not correlated with behavioral parameters at the individual level. Therefore, the behavioral attenuation observed following NAC treatment should be interpreted as being associated with, rather than definitively caused by, modulation of antioxidant pathways. This distinction is particularly relevant because NAC may exert effects through multiple mechanisms, and the present experimental design does not allow their individual contributions to be separated.

Despite these findings, several limitations of the present study should be acknowledged. First, the same zebrafish underwent multiple sequential behavioral tests, which may have influenced subsequent behavioral responses despite the resting intervals provided between assays.

Second, all fish were subjected to a five-day social isolation protocol prior to behavioral testing, and social isolation itself may affect anxiety-like behavior, social interaction, aggression-like responses, and oxidative stress parameters, potentially acting as a confounding factor.

Third, the present study employed an acute immersion-based caffeine and NAC exposure model, which does not fully replicate chronic or oral caffeine consumption in humans. However, this approach is widely used in zebrafish behavioral pharmacology to assess rapid neurobehavioral and biochemical responses under controlled conditions. The chosen exposure durations are consistent with previous studies using similar paradigms, supporting the validity of this experimental model.

Fourth, although a sample size of *n* = 10 fish per group is commonly used in zebrafish behavioral studies, no formal a priori power analysis was performed. Therefore, the possibility of limited statistical power cannot be excluded.

Additionally, although changes in SOD and GPx activity suggest the involvement of oxidative stress-related pathways, these biomarkers alone are insufficient to establish oxidative stress as the definitive causal mechanism underlying the observed behavioral alterations. Furthermore, a notable limitation of the present study is the absence of transcriptional analyses to evaluate the mRNA expression levels of key antioxidant genes (such as SOD and GPx via RT-qPCR). While our biochemical assays demonstrated significant alterations in enzymatic activities following caffeine and NAC treatments, measuring enzyme activity alone provides only a functional snapshot, which may be influenced by post-translational modifications or direct substrate availability rather than transcriptional upregulation. Some studies using zebrafish models for assessing antioxidant gene expression profiles under neurotoxic or stress conditions have demonstrated that functional enzymatic changes do not always directly mirror transcriptional activity, emphasizing the need to evaluate mRNA levels to capture complete regulatory mechanisms [54,55].

For instance, investigations into redox-sensitive regulators and antioxidant defenses in *Danio rerio* have established that tracking downstream target genes such as *sod1*, *sod2*, and *gpx* via RT-qPCR provides critical molecular depth regarding how compounds like NAC trigger cellular adaptation. To bridge this gap, future investigations should incorporate molecular profiling [56,57]. Integrating gene expression analyses alongside functional assays would clarify whether pharmacological interventions like NAC drive de novo synthesis of antioxidant machinery or primarily enhance existing redox buffering capacities. Expanding the experimental design to include such genetic markers, alongside lipid peroxidation assessments and GSH/GSSG balance evaluations, will significantly strengthen the mechanistic interpretation and overall robustness of future neurobehavioral findings.

Future studies including additional markers such as lipid peroxidation, catalase activity, GSH/GSSG balance, and brain-specific oxidative stress markers, as well as formal power analysis and expanded experimental designs, would further strengthen the mechanistic interpretation and robustness of the findings.

## 4. Materials and Methods

### 4.1. Ethical Note

Animals were housed and handled in compliance with the recommendations of the European Commission (2007), Directive 2010/63/EU of the European Parliament and of the Council, as well as the provisions established by BECA Decision C3/04.06.2021 regarding the housing, care, and protection of animals used for experimental and scientific purposes. The experimental procedures were approved by the Ethics Committee of the Faculty of Biology, “Alexandru Ioan Cuza” University of Iași (Approval No. 692/11.02.2026).

### 4.2. Animal Maintenance

The study included 60 adult zebrafish, approximately 9–10 months old, from an authorized local breeder and belonging to a wild-type laboratory strain (AB line). The zebrafish used in this experiment were maintained under experimental laboratory conditions of 10 days, in 10 L aquariums, equipped with oxygen pumps and dechlorinated water changed daily, with the following characteristics: temperature 26 ± 1 °C, pH = 7.5, dissolved oxygen 7.20 mg L^−1^, ammonia concentration < 0.004 ppm, and conductivity 500 μS. Housing density was maintained at approximately 1 fish per liter. Fish were fed twice daily with commercial flake food (Tetramin^®^, Tetra, Melle, Germany; ~2–3 mm diameter), and feeding was standardized across all groups and performed at consistent times relative to experimental procedures. The light/dark cycle was maintained at 12:12 h. Sex was identified visually and groups were balanced for male/female ratio. After this period, zebrafish were randomly assigned to six experimental groups (*n* = 10 per group): control, 25 mg/L Caf, 60 mg/L Caf, NAC (1 mg/L), 25 mg/L Caf + NAC (1 mg/L), and 60 mg/L Caf + NAC (1 mg/L). The same fish from each experimental group underwent all behavioral assessments and were subsequently used for biochemical analyses.

### 4.3. Substances and Treatments

Caffeine and NAC solutions were prepared extemporaneously using pure compounds (caffeine, ≥99% purity, and N-acetyl-L-cysteine; Sigma-Aldrich, Merck KGaA, Darmstadt, Germany) and administered to zebrafish via immersion. The selected concentrations were based on the previous literature and pilot observations and were chosen to induce neurobehavioral modulation without causing toxicity or overt physiological impairment. The control group was not exposed to any pharmacological compounds and was maintained under identical environmental and handling conditions as the experimental groups. Immersion-based caffeine exposure has been shown to modulate locomotor activity, anxiety-like behavior, and adenosinergic signaling, while NAC exposure influences oxidative stress balance, neuroinflammatory pathways, and stress-related behavioral responses. Collectively, these findings indicate that immersion is a reliable and non-invasive method for achieving neuroactive exposure to caffeine and NAC in zebrafish models.

NAC uptake via immersion exposure in zebrafish is well documented, as small molecules can be absorbed through the gills and skin, allowing systemic bioavailability and biological activity in behavioral and biochemical assays.

All solutions were prepared in standardized dechlorinated system water under identical conditions across experimental groups, and pH was monitored during preparation. No relevant differences in pH were observed between control and treatment solutions.

### 4.4. Experimental Design

**Figure 8:**
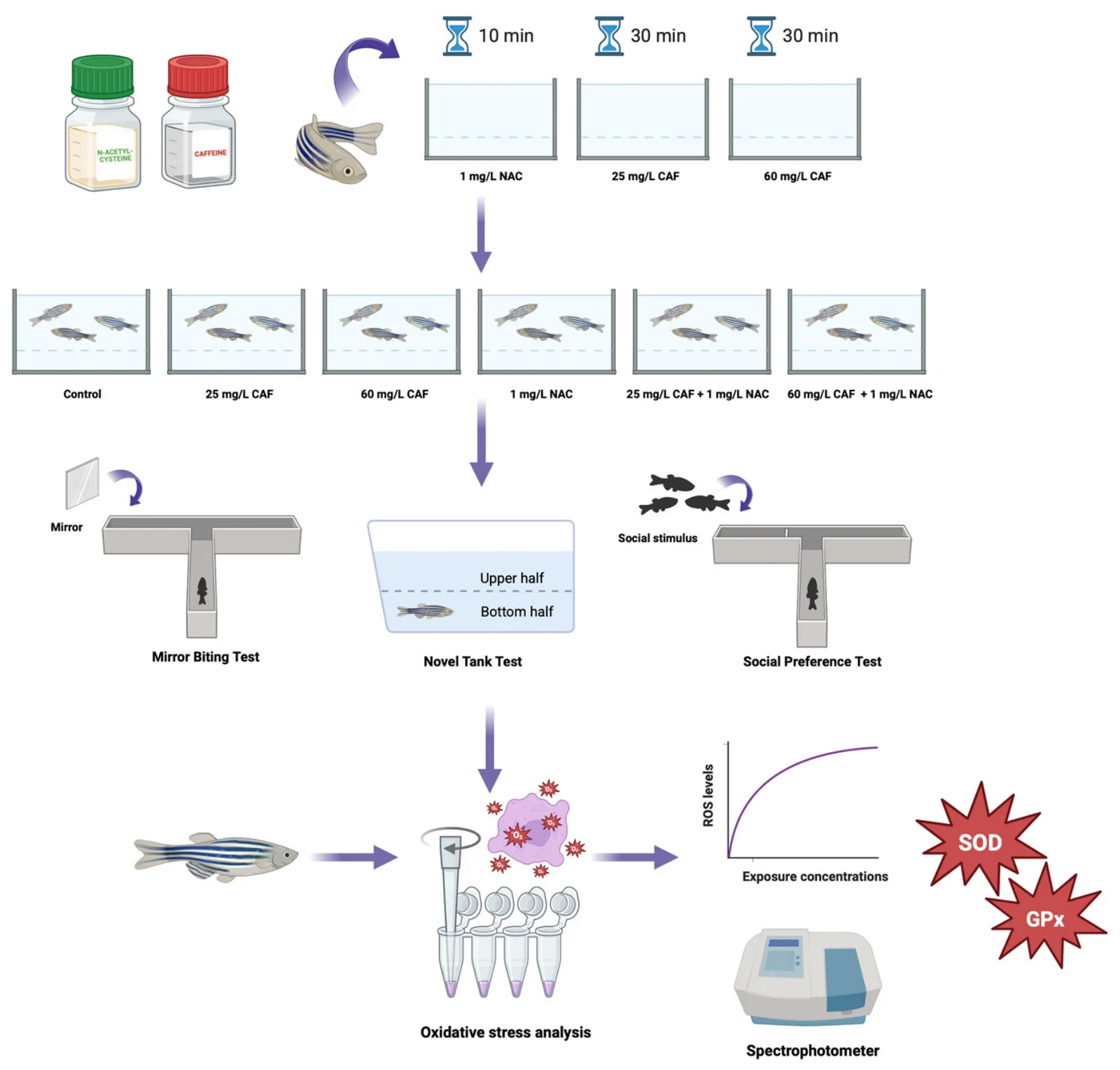
Experimental design illustrating zebrafish exposure to caffeine (CAF) and N−acetylcysteine (NAC), followed by behavioral testing and biochemical analysis. Created in BioRender. Ionescu, C. (2026) https://BioRender.com/qho77oa.

Zebrafish were exposed to NAC and caffeine to investigate the preventive effects of NAC on caffeine-induced anxiety-like behavior, social behavior, and aggression. NAC was administered at a concentration of 1 mg/L via immersion for 10 min (based of ref. [58]), followed by exposure to caffeine at concentrations of 25 mg/L and 60 mg/L for 30 min, based on previous studies [41] (Figure 8). The concentrations were chosen based on previous studies demonstrating behavioral and neuropsychiatric effects in zebrafish. The administration method via immersion was selected to ensure uniform exposure of the fish to the compounds while minimizing handling stress. NAC was applied first to allow sufficient time for its antioxidant and neuromodulatory effects to take place before the anxiogenic and stimulatory effects of caffeine. Following NAC pretreatment, fish were transferred to caffeine-containing water for acute exposure. Fish were randomly assigned to six experimental groups: a control group receiving no treatment, a NAC-alone group (1 mg/L), caffeine-alone groups at 25 mg/L or 60 mg/L, and combination groups in which fish were pretreated with NAC followed by caffeine at either 25 mg/L or 60 mg/L. This design allowed the assessment of both the individual effects of NAC and caffeine, as well as the potential preventive effects of NAC against caffeine-induced behavioral alterations. Behavioral responses were assessed immediately following treatment using established paradigms in zebrafish behavioral neuroscience. All behavioral tests were performed sequentially using the same zebrafish from each experimental group (*n* = 10 per group). The order of testing was as follows: Social Preference Test, Mirror-Biting Test, and Novel Tank Test. To minimize acute handling stress and carryover effects between paradigms, a resting interval of approximately 15 min was allowed between consecutive behavioral tests, during which fish were returned to fresh system water under standard environmental conditions. Following completion of all behavioral assessments, zebrafish were humanely euthanized by cold-water immersion in accordance with EU ethical guidelines, and whole-body samples were immediately collected for biochemical analyses of oxidative stress markers. Social behavior and sociability were assessed in a Social Preference Test (SPT), quantifying the time spent near conspecifics versus an empty compartment in a T-maze configuration. Aggressive behavior was measured using the Mirror Biting Test (MBT), evaluating the frequency and duration of attacks directed toward the fish’s mirror image. Anxiety-like behavior was evaluated using the Novel Tank Test (NTT), which measures exploration patterns, freezing episodes, and vertical locomotion. Behavioral tracking and quantitative analysis were performed using EthoVision XT 14 video tracking software (Noldus Information Technology, Wageningen, The Netherlands), allowing automated measurement of locomotor parameters, freezing duration, social approach, and aggressive interactions. This experimental design allowed us to specifically evaluate the immediate pharmacological effects of NAC and caffeine on zebrafish behavior, without confounding influences from long-term adaptation or metabolic changes.

#### 4.4.1. The Social Preference Test

The social preference test was conducted following a protocol previously established in the literature [59]. The apparatus consisted of a T-maze with two equal-length arms (left and right) and a longer start arm. Consistent with the standardized protocol described in previous studies and to maintain methodological comparability, the social stimulus, consisting of a few conspecifics, was placed in the left arm and separated from the test subject by a transparent PVC screen. Zebrafish were socially isolated for five days prior to testing using opaque white dividers between tanks. All experimental groups underwent the same social isolation protocol prior to behavioral testing in order to maintain consistency across experimental conditions. This approach was implemented to standardize baseline social motivation across groups; however, social isolation itself may influence anxiety-like behavior, social interaction, aggression-like responses, and oxidative stress parameters, and this was acknowledged as a limitation of the study.

Exploratory behaviour was recorded for a 5-min session using EthoVision XT version 14 (Noldus Information Technology, Wageningen, The Netherlands) [59,60]. The following behavioural parameters were measured and analyzed: distance moved (cm); velocity (cm/s); time spent in the left arm (s); acceleration (cm/s^2^); mobility state (s); and zone alterations (frequency).

#### 4.4.2. The Mirror-Biting Test

For the mirror test, a mirror was placed in the left arm of the T-maze, and each zebrafish was left for half a minute for accommodation, then individually observed for 4 min, as established in the literature [59]. Images were recorded with a top-view infrared camera and analyzed using EthoVision XT version 14 (Noldus Information Technology, Wageningen, The Netherlands), previously calibrated for this setup. Behavioral parameters analyzed included: distance moved (cm); velocity (cm/s); acceleration (cm/s^2^); time spent near the mirror (s); mobility state (s); turn angle (deg); rotations clockwise (frequency); and rotations counter-clockwise (frequency).

#### 4.4.3. The Novel Tank Test

The novel tank test considers new environments to be anxiogenic for zebrafish, inducing initial occupation of the tank bottom followed by progressive habituation and increased exploration of the upper zone [61,62]. In this study, each zebrafish was individually transferred to a simple tank placed on a white bench, with walls and back covered with dull white paper to facilitate tracking. The tank was filled with a water column suitable for swimming, and no internal or external enrichment was present.

Swimming behaviour and space occupation were recorded for 5 min using a top-view and a side-view camera, and trajectories were analyzed using EthoVision XT version 14 (Noldus Information Technology, Wageningen, The Netherlands). The tank was virtually divided into upper (UP) and bottom (BTM) zones, and the following parameters were measured: distance moved (cm); velocity (cm/s); inactivity time (s); latency to enter the bottom half (s); time spent in the bottom half (s); time spent near the walls (s); clockwise rotations (frequency); and counter-clockwise rotations (frequency).

### 4.5. Biochemical Analysis

Immediately after completion of the final behavioral assessment, zebrafish were humanely euthanized by immersion in ice-cold water (below 5 °C), in accordance with established ethical guidelines for zebrafish research. Whole-body samples from each fish were subsequently collected and stored at −20 °C until oxidative stress parameters were analyzed. For biochemical determinations, each specimen was thawed and homogenized in ice-cold physiological saline (0.90% NaCl). The homogenates were centrifuged at 5500 rpm for 15 min, following Jin’s protocol [63], and the resulting supernatants were collected for further analysis.

SOD was measured using commercially available assay kits (Sigma-Aldrich ) according to the manufacturer’s instructions. Absorbance was recorded with a Specord 210 Plus spectrophotometer (Analytik Jena, Jena, Germany) at specific wavelengths of 450 nm.

GPx activity was determined using commercially available assay kits, following the manufacturer’s protocol. The reaction rate was monitored spectrophotometrically by measuring the decrease in absorbance associated with the oxidation of NADPH at 340 nm. All measurements were performed using a Specord 210 Plus spectrophotometer (Analytik Jena, Jena, Germany). Enzyme activity was expressed relative to protein content in the samples, ensuring comparability between specimens.

### 4.6. Statistical Analysis

The normality of data distribution was assessed using the Shapiro–Wilk test in GraphPad Prism 10.4.1 (San Diego, CA, USA). Statistical differences among groups were analyzed by one-way ANOVA, followed by Tukey’s post hoc multiple comparisons test. The results are presented as mean ± standard deviation (SD), and statistical significance was considered at *p* < 0.05.

The primary behavioral endpoints were predefined based on the objectives of the study and included time spent near the social stimulus (left arm) in the Social Preference Test, time spent near the mirror in the Mirror-Biting Test, and latency to enter the bottom half and time spent in the bottom half in the Novel Tank Test. SOD and GPx activity were considered the primary biochemical endpoints. Additional locomotor and exploratory parameters were analyzed as secondary/exploratory outcomes to provide a broader characterization of behavioral responses.

## 5. Conclusions

The present study demonstrates that acute caffeine exposure at 25 mg/L and 60 mg/L induces significant behavioral alterations in adult zebrafish, affecting social interaction, aggression-like behavior, and anxiety-like behavior. Specifically, caffeine reduced social affiliation, as evidenced by decreased time spent near conspecifics, while simultaneously increasing locomotor activity (distance moved, velocity, acceleration), reflecting a hyperactive or restless behavioral state. Aggression-related parameters, such as time spent near the mirror, directional rotations, and mobility, were also elevated under caffeine exposure. Anxiety-like responses were pronounced in the Novel Tank Test, with increased bot-tom-dwelling, thigmotaxis, and exploratory transitions, alongside hyperlocomotion during the test.

Pre-treatment with NAC effectively attenuated caffeine-induced hyperlocomotion, aggression-like behavior, and anxiety-like behaviors, as reflected by reductions in velocity, acceleration, mobility, directional rotations, bottom-dwelling, and thigmotaxis. However, NAC did not restore social affiliation, indicating a differential effect of NAC across the behavioral domains assessed in the present study. Thus, the attenuation of locomotor, aggression-like, and anxiety-like responses was not accompanied by recovery of caffeine-induced alterations in social preference.

Biochemically, caffeine exposure was associated with altered antioxidant enzyme activity, including increased SOD activity, with significant changes in both SOD and GPx activities. NAC, alone and in combination with caffeine, also modulated SOD and GPx activities, indicating an alteration of enzymatic antioxidant defenses under the experimental conditions. However, these findings do not by themselves establish oxidative stress as a causal or “key mediator” of the observed behavioral effects, but rather indicate modulation of antioxidant defense systems.

The findings collectively highlight that NAC attenuated several behavioral alterations induced by acute caffeine exposure, particularly hyperlocomotion, aggression-like responses, and anxiety-like behavior, while its effect on caffeine-induced impairment of social preference was limited. The accompanying changes in SOD and GPx activities further demonstrate that both caffeine and NAC influenced enzymatic antioxidant responses.

These results reinforce the utility of zebrafish as a translational model for studying the neurobehavioral and biochemical impacts of psychoactive compounds and evaluating potential interventions. Future studies should explore the mechanistic underpinnings of caffeine-induced social avoidance and investigate additional or complementary interventions that could more fully restore social behavior while mitigating hyperactivity, aggression, and oxidative stress.

## Data Availability

Data is contained within the article.

## Supplementary Materials

The following supporting information can be downloaded at: https://www.mdpi.com/article/10.3390/ph19091419/s1. File S1. Additional Results.
